# In a niche‐neutral continuum, a set of theoretical models in a metacommunity operates simultaneously in patchy habitats

**DOI:** 10.1002/ece3.9754

**Published:** 2023-02-22

**Authors:** Takayuki Yunoki

**Affiliations:** ^1^ Centro de Investigación de Recursos Acuáticos (CIRA) Universidad Autónoma del Beni Trinidad Beni Bolivia

**Keywords:** ecology, evolution, individual‐based model, niche‐neutral continuum, spatially‐explicit synthesis

## Abstract

This study investigated the origins and maintenance of biodiversity by integrating ecological and evolutionary mechanisms into a spatially‐explicit synthesis between niche‐based processes and neutral dynamics (ND). An individual‐based model on a two‐dimensional grid with periodic boundary conditions was used to compare a niche‐neutral continuum induced in contrasting spatial and environmental settings while characterizing the operational scaling of deterministic‐stochastic processes. The spatially‐explicit simulations revealed three major findings. First, the number of guilds in a system approaches a stationary state and the species composition in a system converges to a dynamic equilibrium of ecologically‐equivalent species generated by the speciation–extinction balance. This convergence of species composition can be argued under a point mutation mode of speciation and niche conservatism due to the duality of ND. Second, the dispersal modes of biota may affect how the influence of environmental filtering changes across ecological–evolutionary scales. This influence is greatest in compactly‐packed areas within biogeographic units for large‐bodied active dispersers, such as fish. Third, the species are filtered along the environmental gradient and the coexistence of ecologically‐different species in each local community in a homogeneous environment is allowed by dispersals in a set of local communities. Therefore, the ND among the single‐guild species, extinction–colonization trade‐off among species of similar environmental optima and different levels of specialization, and mass effect, such as weak species–environment associations, operate simultaneously in patchy habitats. In spatially‐explicit synthesis, characterizing where a metacommunity falls along a niche‐neutral continuum is too superficial and involves an abstraction that any biological process is probabilistic; therefore, they are dynamic–stochastic processes. The general patterns observed in the simulations allowed a theoretical synthesis of a metacommunity and explained the complex patterns observed in the real world.

## Graphical abstract

This study investigated the origins and maintenance of biodiversity by integrating ecological and evolutionary mechanisms into a spatially‐explicit synthesis between niche‐based processes and neutral dynamics. An individual‐based model on a two‐dimensional grid with periodic boundary conditions was used to compare a niche‐neutral continuum induced in contrasting spatial and environmental settings while characterizing the operational scaling of deterministic‐stochastic processes.

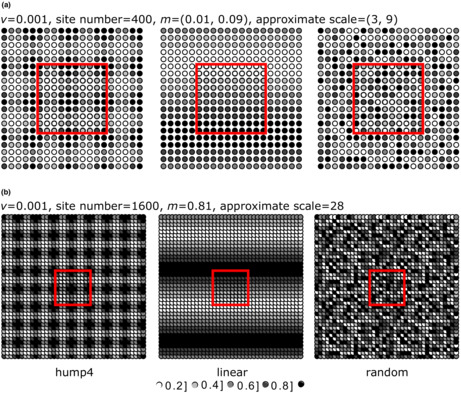

## 1 INTRODUCTION

Ecologists have traditionally relied on deterministic processes, such as environmental niches, to explain the coexistence of species using a stable equilibrium among species populations (Gause, 1934; Hutchinson, 1957). Recently, ecologists have recognized the role of stochastic processes. For example, neutral dynamics (ND) is derived from the primary assumption of per capita niche equivalence among individuals and saturation of diversity in communities because of ecological drift, random speciation, and migration. Under ND, the coexistence of species is explained by the dynamic equilibrium of ecologically‐equivalent species within a set of local communities (Durrett & Levin, 1996; Hubbell, 2001). In contemporary ecology, a modeling approach to integrate ecological–evolutionary mechanisms into a synthesis between niche‐based processes and ND is a promising perspective for explaining the origin and maintenance of biodiversity (Hubbell, 2001; Jabot et al., 2008; Janzen et al., 2015; Leibold & Mcpeek, 2006; Munoz et al., 2018).

Individual‐based models are one approach for implementing such a synthesis. Such models are mostly implemented in spatially‐implicit conjectures based on a hierarchical concept inspired by a mainland–island system, where the extinction of a metacommunity is balanced by speciation through panmixis. However, local community extinction is balanced by immigrants from a metacommunity. Under ND in a metacommunity, species richness and relative species abundance at equilibrium are completely driven by the metacommunity size and speciation rate. The prediction of these measures in a local community is based on two parameters: local community size and dispersal rate (Etienne, 2005, 2007; Hankin, 2007; Hubbell, 2001; Munoz et al., 2007).

During several spatially‐implicit integrations of niche‐based processes and ND, a metacommunity undergoes evolutionary dynamics, whereas each local community follows an extinction–immigration balance with possible environmental filtering (Jabot et al., 2008; Janzen et al., 2015; Munoz et al., 2018). A reference species pool must be flexibly defined, otherwise the prediction of the regional pool is challenging (Jabot et al., 2008; Munoz et al., 2018). This makes a strong assumption about panmixis in the metacommunity (Janzen et al., 2015). The spatially‐implicit approach allows for the decoupling evolutionary dynamics in a metacommunity and ecological processes in each local community (Munoz et al., 2018).

A spatially‐explicit approach implemented on the lattice offers an opportunity to develop a unified framework that addresses the role of dispersals in a metacommunity more realistically and integrates ecological and evolutionary dynamics into a synthesis between niche‐based processes and ND. For instance, when modeling ND in a portion of an infinite system, species richness at equilibrium in a metacommunity is derived from metacommunity size and different combinations of speciation rates and dispersal kernels (Rosindell et al., 2008). Furthermore, when implementing the neutral model with the nearest neighboring communities, different combinations of speciation and dispersal rates (i.e., low speciation and low dispersal rates vs. high speciation and high dispersal rates) yield the same approximate scales at which individuals diffuse before speciation in a metacommunity (Cencini et al., 2012).

However, to reconcile niche‐based processes and ND in a spatially‐explicit approach requires acknowledging that niche‐based processes are derived from the per capita niche differences among species along the environmental gradient. Competitive exclusion among species in each local community (Gause, 1934) and environmental filtering of species along the environmental gradient may lead to a stable equilibrium of ecologically‐different species among patchy heterogeneous habitats (Hutchinson, 1957; Leibold & Mcpeek, 2006). ND is derived from per capita niche equivalence among species, allowing speciation and dispersal limitations across spatiotemporal scales. The speciation–extinction balance may eventually drive a dynamic equilibrium of ecologically‐equivalent species within the patchy habitat of a homogeneous environment in their exclusive network of local communities (Economo & Keitt, 2008; Thompson et al., 2020). An efficient coalescence method can no longer ignore the neutral assumption in spatially‐explicit synthesis, and such syntheses can only be simulated using forward methods (Rosindell et al., 2008; Thompson et al., 2020).

To model an area sampled from a larger system, model communities must be sampled from a larger system where the environmental gradient repeats continuously across opposite sides of the model communities with periodic boundary conditions. In this model, the approximate scale expected under ND in a portion of an infinite system sets the distance to render the influence of evolutionary dynamics from the outside of model communities an independent notion. In addition, the approximate scale and size of model communities tend to control how compactly‐packed they are within an independent biogeographic unit. Based on the spatial variation in biodiversity expected under ND, the relative role of ND appears to increase in the model communities comprising several independent biogeographic units because speciation can contribute more to the species richness of ecologically‐equivalent species.

A modeling approach was introduced to integrate ecological and evolutionary dynamics into a spatially‐explicit synthesis between niche‐based processes and ND. An individual‐based model on a two‐dimensional grid with periodic boundary conditions is used to compare a niche‐neutral continuum induced in contrasting spatial and environmental settings. In addition, the model characterizes the spatiotemporal scales at which niche‐based processes drive a stable equilibrium among ecologically‐different species and ND drives a dynamic equilibrium of ecologically‐equivalent species. Three hypotheses were tested starting with nearly identical ranges of diversity in contrasting spatial and environmental settings.

First, the number of guilds (i.e., species groups that have different environmental niches) in a system initially reaches a stationary state; then, the species composition in a system eventually converges to a dynamic equilibrium of ecologically‐equivalent species generated by the speciation–extinction balance. Second, the relative role of ND increases in areas comprising several independent biogeographic units through speciation. Third, the species were filtered along the environmental gradient, and the species of each guild were packed within the patchy habitat of a homogeneous environment in their exclusive network of local communities. According to the results, the last two hypotheses appeared to be context‐sensitive; however, the general patterns observed in the simulations provided intuitive explanations of a larger array of biodiversity patterns observed in the real world.

## 2 MATERIALS AND METHODS

### 2.1 Simulation description

In the simulations, individuals and species from different guilds occupied different environmental niches. Individuals and species from the same guild occupy the same environmental niches. The niche‐based processes were derived specifically from the differences in per capita probabilities of immigration success of individuals to each local community. The environmental niches did not affect the per capita birth and death rates of individuals in each local community. The ND was derived from the primary assumption of per capita niche equivalence among individuals, allowing speciation and dispersal limitations across spatiotemporal scales.

### 2.2 Simulation mechanics

**FIGURE 1:**
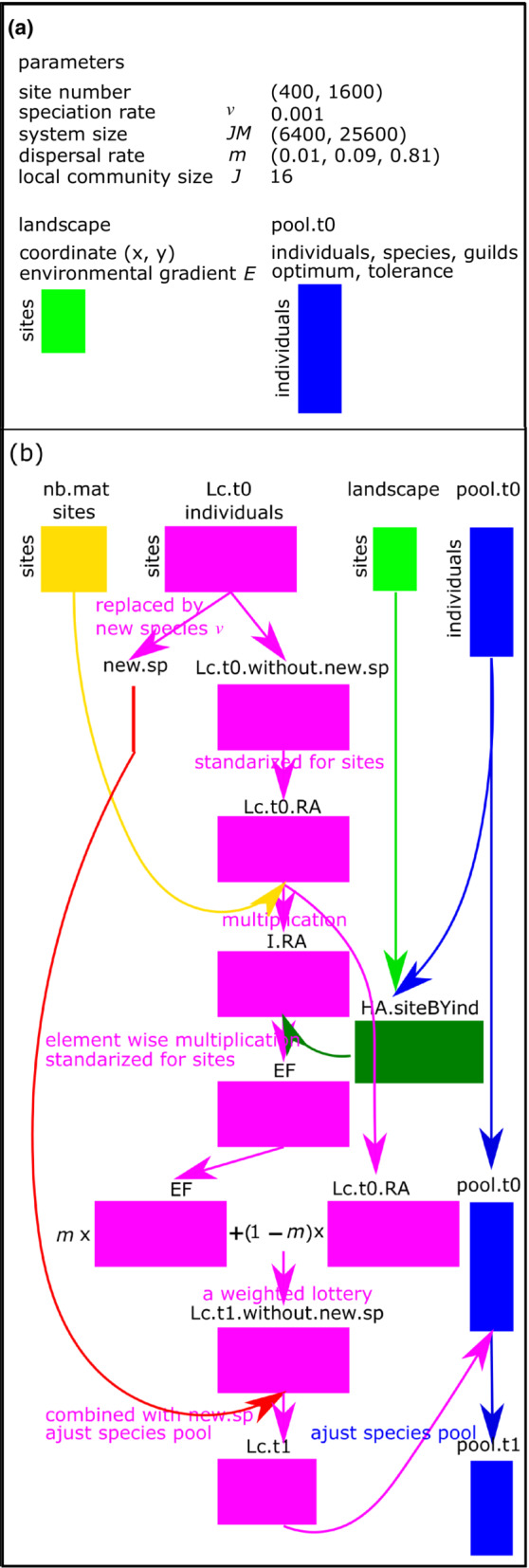
A workflow script illustrating an individual‐based model. (a) Parameters, (b) first time step.

Figure 1a,b provide a schematic overview of the simulation mechanics and algorithm. The simulations are initiated by the following parameters for a neutral model: two site level numbers (400 and 1600), a fixed speciation rate (*ν* = 0.001), with point mutation mode of speciation and niche conservatism, two system size levels (JM = 6400 and 25,600), three levels of dispersal rate (*m* = 0.01, 0.09, and 0.81), and a fixed local community size (*J* = 16). Based on the site number, a square grid of local communities was specified with coordinates (*x*, *y*) and environmental gradient *E* in the matrix **landscape.** An initial species pool was specified in the matrix **pool.t0** using the following parameters: individual ID, species ID, guilds ID, and their environmental niche (i.e., environmental optimum and tolerance) (Figure 1a).

A weight matrix for the nearest neighboring communities with a spatial weight of 1/8 was generated in a grid onto the torus (matrix **nb.mat**). The system individuals were randomly assigned to their initial locations (matrix **LC.t0**). At each time step, all individuals were removed and replaced by new species (matrix **new.sp**) with a probability *ν*. The matrix of the remaining individuals (matrix **LC.t0.without.new.sp**) was divided by the row sum for each site (matrix **LC.t0.RA**) and multiplied by the matrix **nb.mat** to generate a set of immigration success probabilities of individuals to each local community under per capita equivalence (matrix **I.RA**). Matrix **I.RA** was multiplied element‐wise by a matrix of habitat associations of individuals to sites (matrix **HA.siteBYind**) and divided by the row sum for each site to generate a set of probabilities of the immigration success of individuals to each local community as a function of their environmental niches (matrix **EF**).

The assigned weights in matrix **HA.siteBYind** depended on the value of the probability density function for normal distribution, where the mean is the environmental optimum of individuals and the standard deviation is the tolerance of individuals for the local environment of sites divided by the row maximum for each site. The parents of each local community were chosen using a corresponding probability vector in *m* × matrix **EF** + (1 − *m*) × matrix **LC.t0.RA** as the sum of immigrant (left term) and local birth (right term) parents (matrix **LC.t1.without.new.sp**).

Finally, the matrix **LC.t1.without.new.sp** was combined with the matrix **new.sp** and the extinct lineages of individuals were removed (matrix **LC.t1**). In addition, matrix **pool.t0** was adjusted for the species pool (matrix **pool.t1**). The new species were identified by the codes of ancestor individuals and the number of time steps in matrix **LC.t1**, and identified by these codes in matrix **pool.t1** for individual and species levels. The descendants from a common ancestor individual were identified by the same code, and each lineage was grouped in a single column in matrix **LC.t1** and a single row in matrix **pool.t1**. Matrix **pool.t1** and matrix **LC.t1** provided the input data for the next time step (Figure 1b).

### 2.3 Experimental design

**FIGURE 2:**
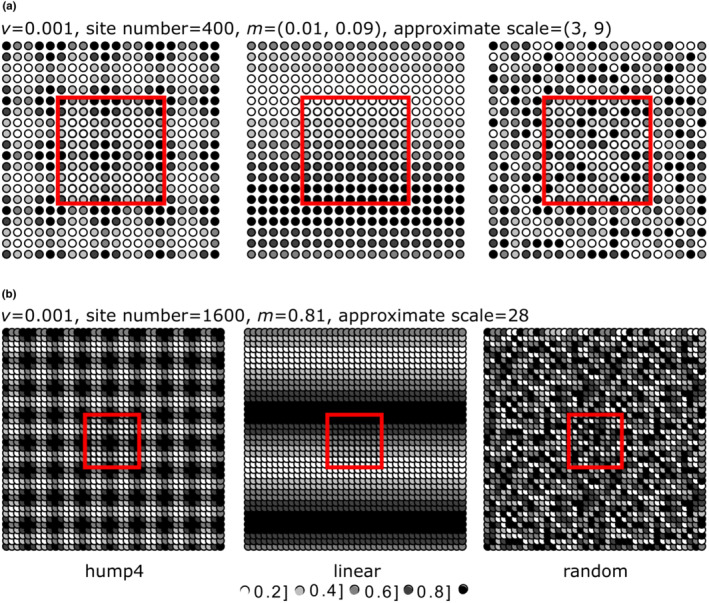
Setting the contrasting spatial scales and environmental structures of model communities. The simulations were initiated by a fixed speciation rate, *ν* = 0.001, for the nearest neighboring communities. The model communities were a grid of 10 × 10 local communities in the system center. (a) The model communities were sampled from a small system simulated over a grid of 20 × 20 local communities for two lower dispersal rates, *m* = (0.01, 0.09). The approximate scales were three for *m* = 0.01 and nine for *m* = 0.09. (b) The model communities were sampled from a large system simulated on a 40 × 40 grid of local communities for the maximum dispersal rate, *m* = 0.81. The approximate scale of the large system was 28. In both systems, the environmental gradient of model communities varied in three structures: four humps, linear structure, and random structure and repeated continuously across the opposite sides of model communities.

The model communities of three spatial scales were set relative to an independent biogeographic unit. The model communities were a grid of 10 × 10 local communities in the center of the system. For a neutral model with nearest neighboring communities and a fixed speciation rate (*ν* = 0.001), the approximate scales were three for dispersal rate *m* = 0.01, nine for *m* = 0.09, and twenty‐eight for *m* = 0.81 (Cencini et al., 2012). In the simulations, dispersal rates were used to set the system sizes, to render the influence of external evolutionary dynamics of model communities as an independent factor, and to set how compactly the model communities were packed within independent biogeographic units. The model communities were sampled from a small system simulated over a grid of 20 × 20 local communities for two lower dispersal rates (*m* = 0.01 and 0.09) (Figure 2a) and from a large system simulated on a 40 × 40 grid of local communities for the highest dispersal rate (*m* = 0.81) (Figure 2b).

Within the biogeographic units, the model communities sampled from the large system (*m* = 0.81) were packed nine times more compactly than those sampled from the small system (*m* = 0.01). While a real ecosystem may be vast, extinction may be balanced by the minimum speciation rate (Bell, 2003). Ecologists frequently study biogeographic units packed much more compactly. However, to date, the computation of larger systems with lower speciation rates has not been possible.

### 2.4 Setting the model communities of three environmental structures

Previous studies have suggested that the dispersal rates of organisms and the spatial structures of the environment have complex effects on the species–environment relationships and the spatial distribution of species. However, there is still no established statistical analysis method to quantify this complex effect accurately (Smith & Lundholm, 2010; Sokol et al., 2017).

In combination with spatial scales, the environmental values of the model communities varied in three contrasting structures: four humps, linear structures, and random structures. In a random environment, there is no correlation between the environmental values of different local communities, and the variance was assumed to be constant. In a four‐hump environment, the variance between the environmental values of different local communities depends only on the distance between local communities. In a linear environment, the spatial structures are more complex. The variance between the environmental values of different local communities depends on the distance and the direction between local communities. In all cases, the environmental values for each local community were bounded between 0 and 1, the number of local communities presenting each environmental value was approximately equal, and the environmental gradient was repeated continuously across the opposite sides of the model communities. Therefore, in the small system, the environmental gradient *E* presented three structures: random, one wave, and 16 humps (Figure 2a). In the large system, the environmental gradient quadrupled and presented three structures: random, two waves, and 64 humps (Figure 2b).

### 2.5 Creating a niche‐neutral continuum in contrasting spatial and environmental settings

To evaluate the hypotheses, simulations were initialized from nearly identical ranges of diversity in contrasting spatial and environmental settings. This transformed the initial species pool of the system from species without niche differences to species with strong niche differences.

The initial species pool of the system varied according to the number of guilds *g*, environmental niches, and population sizes of guilds. The environmental optimum of guilds was assigned following a uniform distribution ranging from 0 to 1 and the tolerance of guilds was assigned ranging between 0 and 10. The population sizes of guilds were generated by splitting JM at *g* − 1 points. These points were *g*‐1 integers sampled from a uniform distribution between 1 and JM − 1, and sorted according to their values. In the small system, simulations for each combination of dispersal rate and environmental structure were initialized from the same set of five levels of *g* (*g* = 1, 8, 40, 160, 500), five environmental niches, and five guild population sizes, except for the case of *g* = 1 (i.e., the simulations were initiated from 25 environmental niches of one guild). In a large system, the initial species pool of the small system quadrupled. The simulations in each environmental structure started from the same set of five levels of *g* and five environmental niches of guilds. However, only one case of guild population size was explored because the large system analysis was computationally intensive and time‐consuming. Thus, a total of 825 scenarios were simulated with 750 (2 × 3 × 4 × 5 × 5 + 2 × 3 × 1 × 25) in the small system with two lower dispersal rates and 75 (1 × 3 × 5 × 5) in the large system with the highest dispersal rate.

The simulation of the individual‐based model was performed in R version 3.6.3 (R Core Team, 2019) using the packages spdep (Bivand et al., 2019), vegan (Oksanen et al., 2019), principal coordinates of neighbor matrices (PCNM) (Legendre et al., 2012), randomizr (Coppock et al., 2019), reshape2 (Wickham, 2017), *dplyr (*Wickham et al., 2019), entropart (Marcon & Hérault, 2019), packfor (Dray et al., 2016), pgirmess (Giraudoux et al., 2018), and ape (Paradis et al., 2019). The R resources are available at https://github.com/takayukiyunoki/spatialIBM.

### 2.6 Hypothesis test of convergence to a dynamic equilibrium

In the initial simulations, the number of guilds in the system first reached a stationary state; then, the species richness achieved a dynamic equilibrium through the speciation–extinction balance. Furthermore, setting the same seeds in a random number generator, the simulation outcome from monodominance guilds (i.e., all individuals of each guild were of a single species) converged to the simulation outcome from an infinite diversity of guilds (i.e., all individuals of each guild were different species) when all species of each guild originated from different ancestral individuals (hereafter convergence time). The simulations reported here started with the monodominance guilds.

### 2.7 The relative role of ND in areas comprising several independent biogeographic units

The relationship between the initial species pools and simulated species compositions in contrasting spatial and environmental settings was compared.

The species‐neutral and functional diversities were calculated by considering species abundances for the model communities at the convergence time. This provides the effective number of species in Simpson's index and Rao's quadratic diversity. Functional diversity was based on the similarity matrix between pairs of species and calculated as the overlapping percentage of Gaussian functions (Marcon & Hérault, 2015). The functional uniqueness and redundancy proposed by Ricotta et al. (2016) were used to represent the relative roles of the niche‐based process and the ND.

As noted above, once the parameters of the neutral model and environmental gradient in the system were fixed; the species compositions from different species‐neutral guild diversities converged to a dynamic equilibrium. Therefore, when considering all individuals of each guild as a single species, the simulation outcomes depend on the initial functional diversity of the system. The relationship between the initial Rao's quadratic diversity and the functional redundancy of the model communities was compared across ecological–evolutionary scales in each environmental structure.

### 2.8 Species of each guild within the patchy habitat of a homogeneous environment

To test the species–environment relationships and species turnover in patchy habitats, the species abundance variation in the model communities at convergence time was partitioned using two sets of predictors: environmental and spatial variables, based on redundancy analysis (Borcard et al., 2011; Peres‐Neto & Legendre, 2010). Autocorrelation analysis (Diniz‐Filho et al., 2012) was used to confirm the association of environmental components with niche‐based processes and spatial components with ND. Model communities that produced only one guild and those that produced two or more guilds were treated differently. The findings of variation partitioning and autocorrelation analysis were compared across ecological–evolutionary scales in each environmental structure.

In general, the variation partitioning and autocorrelation analyses followed previously described methods (Yunoki et al., 2017, 2018, 2019; Yunoki & Torres, 2016). In variation partitioning, the species abundance data were Hellinger‐transformed before analysis. Previous studies have clearly shown the problem of using an original environmental variable to model the unimodal responses of species when niche differences among species are strong (Smith & Lundholm, 2010; Sokol et al., 2017). Therefore, the first‐ and second‐order orthogonal environmental variables were used to model the unimodal responses of species (Borcard et al., 2011). The standard forward selection procedure was used because species from the same guild had the same environmental niches and may have similarities in their environmental and spatial associations (Borcard et al., 2011; Peres‐Neto & Legendre, 2010). The proportion of species abundance variation explained by environmental, purely environmental, spatial, purely spatial, and total explained variation (i.e., environmental and spatial) components, which were obtained using the adjusted coefficient of determination. The relative proportions of species abundance variation explained by environmental and purely spatial components in the total explained variation were used to estimate the relative roles of niche‐based processes and ND. The significance of these components was estimated with a randomization test against the null hypothesis of no relationship, applying an alpha value of 0.05 (a one‐tailed test in the upper tail). Tests based on environmental components are known to be biased when both species and environment are spatially structured. Therefore, statistical tests of species–environment relationships were based on pure environmental components.

If at least two guilds coexisted in the model communities and the purely environmental component was significant, the hierarchical guild structure was identified by the *k*‐means partitioning of linear combination scores (scaling 1) and simple structure index criterion. If the hierarchical guild structure was significant, PCNM was constructed to model the spatial structures within and among patchy habitats in the residual variation of species composition between sites.

The functional uniqueness and redundancy of model communities were compared to the relative proportions of species abundance variation explained by environmental and purely spatial components in the total explained variation of the overall model. The total explained variation and relative proportions between the overall and hierarchical models were compared. Furthermore, the number of guilds coexisting in the model communities was compared with the number of habitat types identified by the hierarchical guild structure.

In autocorrelation analysis, the species abundances under ND would be uncorrelated; Moran's I correlograms revealed similar short‐distance autocorrelation patterns; therefore, the Mantel correlation between matrices of correlation coefficients and correlogram distances among species would be zero. By increasing the influence of niche‐based processes, the abundances within ecologically‐equivalent species would generate a positive correlation and a similar correlogram; therefore, the Mantel correlation between these matrices would be negative (Diniz‐Filho et al., 2012).

In single‐guild model communities, autocorrelation analysis was applied for the species abundances predicted by truly spatial components (i.e., when the spatial or purely spatial components were significant but the environmental or purely environmental components were not) and false environmental components (i.e., when the purely environmental component was significant). In model communities with two or more guilds, this method was applied to the true environmental component (i.e., when the purely environmental component was significant) of the overall model and the spatial structures found by the hierarchical model in each environmental context. The hierarchical model is represented by the hierarchical guild structure subtracted from the full hierarchical model when the spatial structures within and among patchy habitats in residual variation were significant. The correlograms were calculated using the number of distance classes computed using the Sturges method in the overall model and three or two distance classes in each environmental context. In all cases, the Mantel correlation was tested against the alternative hypothesis of less than zero by applying an alpha value of 0.05. Appendix S1 presents the summary statistics of the scenarios.

## 3 RESULTS

The six scenarios in the small system possessed an environmental tolerance of nearly zero (<0.0052) and were excluded from the analysis. The results presented herein were based on 819 simulation outcomes.

**FIGURE 3:**
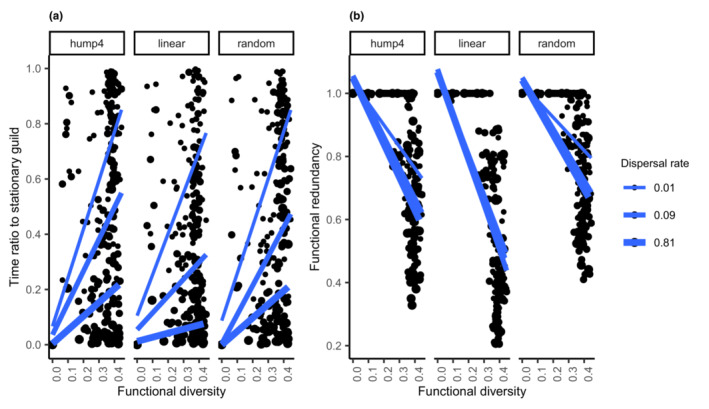
Both ecological differences and equivalences among species were operational at different spatiotemporal scales. (a) System convergence; scatter plots showing the time ratio to stationary guild number in the convergence time of species richness. (b) The relationship between the initial Rao's quadratic diversity and functional redundancy of model communities was compared across ecological–evolutionary scales in each environmental structure. The lines of best fit were added together.

Across the scenarios, the number of guilds in the system first reached a stationary state, and the species richness converged to a dynamic equilibrium through the speciation–extinction balance. Notably, the number of guilds reached a stationary state faster at higher dispersal rates (Figure 3a).

### 3.1 The relative role of ND in areas comprising several independent biogeographic units

Starting with nearly identical ranges of diversity in contrasting spatial and environmental settings, the functional redundancy of model communities appeared to increase in areas consisting of many independent biogeographic units, most markedly in the random environment and four humps (Figure 3b).

### 3.2 Species of each guild within the patchy habitat of a homogeneous environment

**FIGURE 4:**
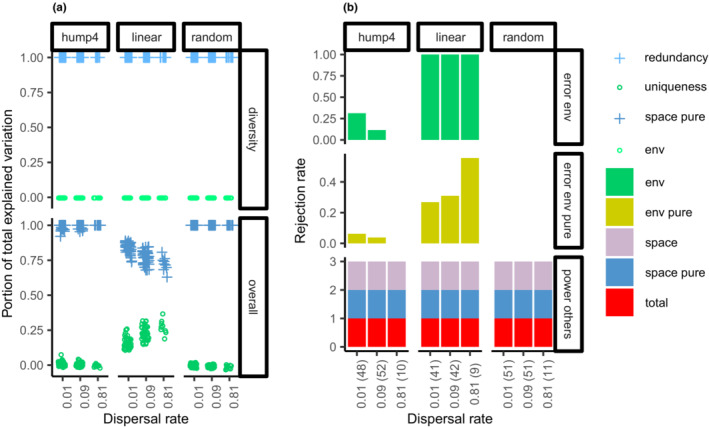
Summary statistics of the model communities that resulted in only one guild were compared across ecological–evolutionary scales in three environmental structures. (a) Functional uniqueness and redundancy were compared to the relative proportions explained by environmental and purely spatial components in total explained variation. (b) Type I error rate of environmental and purely environmental components and power rate of other components. The number of model communities is provided within parentheses on the x‐axis.

In single‐guild model communities, the functional uniqueness was zero. However, the relative proportion explained by environmental components in the total explained variation was positively biased in the linear environmental gradient, markedly at the maximum dispersal rate in areas packed within a biogeographic unit (Figure 4a). The Type I error of the purely environmental component was inflated in these scenarios. The spatial and purely spatial components were always significant (Figure 4b).

**FIGURE 5:**
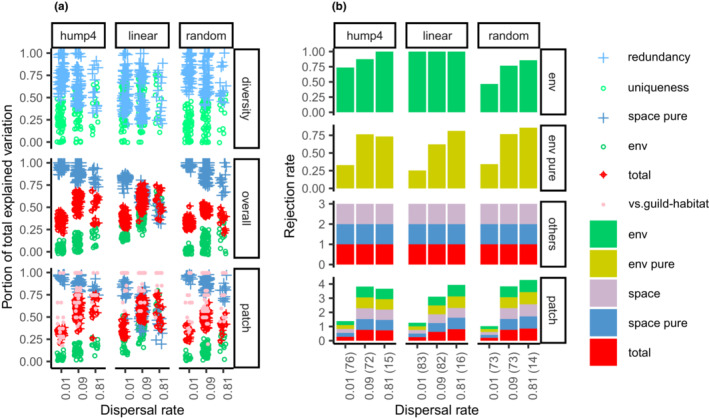
Summary statistics of the model communities that resulted in at least two guilds were compared across ecological–evolutionary scales in three environmental structures. (a) Functional uniqueness and redundancy were compared to the relative proportions explained by environmental and purely spatial components in the total explained variation of the overall model. The total explained variation and relative proportions were compared between overall and hierarchical models. The portions explained in the hierarchical model were calculated for scenarios in which the hierarchical guild structure was significant. The number of guilds that coexisted in model communities (vs. guild habitat) was compared with the number of habitat types identified by hierarchical guild structures. (b) Power rate of all components in overall and hierarchical models. The number of model communities is provided within parentheses on the x‐axis.

In the model communities that resulted in two or more guilds, functional uniqueness and redundancy presented opposite patterns across ecological–evolutionary scales. The functional uniqueness increased in areas packed within biogeographic units that were approximately represented by the relative environmental component in the total explained variation of the overall model (Figure 5a). The power of the purely environmental components increased in these scenarios. The spatial and purely spatial components were always significant (Figure 5b).

If the purely environmental component of the overall model was significant, the hierarchical guild structure and spatial structures found within and among the patchy habitats were usually significant (Figure 5b). The total explained variation and the relative proportions of variation explained by environmental and purely spatial components were similar between the overall and hierarchical models. However, the number of guilds coexisting in model communities was often larger than the number of habitat types identified by the hierarchical guild structure (Figure 5a).

**FIGURE 6:**
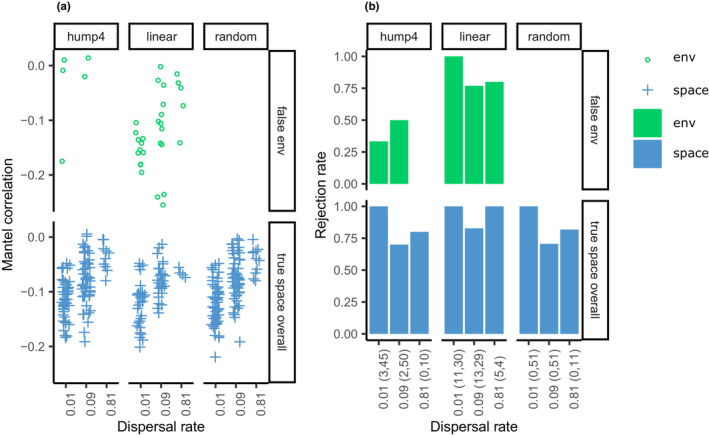
Performance of autocorrelation analysis in the model communities that resulted in only one guild. (a) Mantel correlation of false environmental and true spatial components. (b) Mantel test against the alternative hypothesis of less than zero. The numbers of false environmental and true spatial components are provided within parentheses on the x‐axis.

In single‐guild model communities, the Mantel correlations of the true spatial component approached zero in areas packed within biogeographic units (Figure 6a). The Mantel correlations were always significant in areas encompassing independent biogeographic units and only disappeared for some scenarios in areas packed within biogeographic units (Figure 6b). The Mantel correlations of false environmental components also approached zero in areas packed within biogeographic units (Figure 6a); however, they were often significant and did not correctly control false detection in a linear environmental gradient (Figure 6b).

**FIGURE 7:**
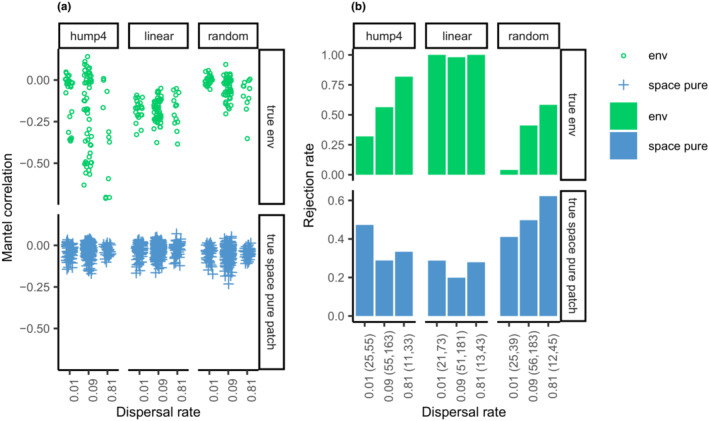
Performance of autocorrelation analysis in the model communities that resulted in at least two guilds. (a) Mantel correlation of true environmental component in the overall model and true purely spatial component in each environmental context. (b) Mantel test against the alternative hypothesis of less than zero. The number of true environmental components in the overall model and the number of environmental contexts in the hierarchical model are provided within parentheses on the x‐axis.

In model communities with at least two guilds, the Mantel correlations of the true environmental component departed negatively in areas packed within biogeographic units (Figure 7a) and generated significant patterns (Figure 7b). The Mantel correlations of the spatial structures found using the hierarchical model in each environmental context were often significant (Figure 7b).

## 4 DISCUSSION

A spatially‐explicit approach implemented on the lattice offers an opportunity to develop a unified framework that allows us to address the role of dispersals more realistically in a metacommunity and integrate ecological and evolutionary dynamics into a synthesis between niche‐based processes and ND. The spatiotemporal scales on which niche‐based processes drive a stable equilibrium among ecologically‐different species and ND drives a dynamic equilibrium of ecologically‐equivalent species, specifically, three hypotheses were evaluated by comparing a niche‐neutral continuum induced in contrasting spatial and environmental settings. Except for the first hypothesis, the other two hypotheses were context‐sensitive.

The first hypothesis was that the number of guilds in a system initially reached a stationary state; then, the species composition eventually converged to a dynamic equilibrium generated by the speciation–extinction balance. Spatially‐explicit simulations reveal that the first hypothesis is true and the number of guilds in the system first reaches a stationary state. The convergence of species compositions can be argued under a point mutation mode of speciation and niche conservatism. Convergence to dynamic equilibrium from any initial diversity was suspected in a neutral spatially‐explicit conjecture (Hubbell, 2001). The neutral model, specifically for a network of local communities, is dual to the system of coalescing random walkers with an annihilation rate (Thompson et al., 2020). Based on this duality of the neutral model, the simulations starting from monodominance guilds achieved a dynamic equilibrium at convergence time because all individuals in each guild were coalesced or annihilated.

Previous studies have found it difficult to confidently confirm the convergence of species composition in forward methods. Moreover, stationarity is argued usually based on asymptotic condition (Hankin, 2007; Rosindell et al., 2008), and little attention has been paid to the detailed changes of species properties in the simulation outcomes (Munoz et al., 2018). In addition, this asymptotic approach, although intuitive, contains erroneous abstraction that all biological processes are probabilistic; therefore, they become dynamic–stochastic processes.

The second hypothesis is supported by a range of key parameters and the context implemented in this study. The functional redundancy of the model communities increased in areas with several separate biogeographic units, indicating that the relative role of ND increased via speciation. This prediction was examined exclusively from the spatial variation of biodiversity expected under ND (Cencini et al., 2012). However, the complex patterns observed in the simulations highlighted its sensitivity.

The third hypothesis was that the species were filtered along the environmental gradient and that the species of each guild were packed within a patchy habitat in a homogeneous environment in their exclusive network of local communities. The results of the spatially‐explicit simulations were interesting, albeit unexpected. The functional uniqueness and relative proportion explained by environmental components increased in the model communities that resulted in two or more guilds. The influence of environmental filtering was greatest at the maximum dispersal rate in areas packed within biogeographic units. The total explained variation and the relative proportions explained by environmental and purely spatial components were similar between the overall and hierarchical models. However, the number of guilds coexisting in model communities was often larger than the number of habitat types identified by the hierarchical guild structure. ND drives a dynamic equilibrium of ecologically‐equivalent species in a network of local communities; however, networks of different guilds are not always spatially mutually exclusive. The coexistence of ecologically‐different species in each local community in a homogeneous environment was allowed by dispersals in a set of local communities, and not only ND among the species of a single guild but also the extinction–colonization trade‐off among species of similar environmental optimum and different levels of specialization (i.e., patchy‐dynamics: PD) (Levins & Culver, 1971; Tilman, 1994) as well as the mass effect (ME) (Mouquet & Loreau, 2003) like weak species–environment associations, operated in the spatial structures found within and among patchy habitats.

### 4.1 Theoretical synthesis of a metacommunity

The concept of metacommunity attempts to explain the coexistence of species in a set of local communities. The review of spatially‐implicit models identified four paradigms: PD, species sorting (SS), ME, and ND (Leibold et al., 2004). The first three paradigms break the neutral assumption: PD assumes an extinction–colonization trade‐off among species in a homogeneous environment. Both SS and ME assume a species–environment association. However, dispersal is high in ME; therefore, species may end up in less favorable sites. By contrast, ND assumes per capita equivalence among individuals in a homogeneous environment. Each paradigm focuses on mechanisms derived from distinct assumptions. In the simulations reported here, the species were filtered along the environmental gradient. ND, PD, and weak ME‐like species–environment associations operated in patchy habitats. These paradigms could be synthesized by their assumptions about trade‐offs among species traits along the environmental gradient, except for SS and ME. Dispersal rates have complex effects not only on the richness of ecologically‐equivalent species through evolutionary dynamics (Cencini et al., 2012) but also on the number of ecologically‐different species in model communities at equilibrium (Mouquet & Loreau, 2003). In the classic spatially‐implicit SS‐ME model, the environmental niche only affects the per capita birth and death rates (Mouquet & Loreau, 2003). For this scenario, the maximum dispersal rate may decrease the number of ecologically‐different species and shift the maximum environmental filtering to an intermediate ecological–evolutionary scale (Ng et al., 2009). This was confirmed in this study through complementary simulations. If the environmental niche only affected the per capita birth and death rates, the functional uniqueness of model communities would be greatest at an intermediate scale (Appendix S2a). Although the neutral assumption can be broken by both contexts in the real world, for instance, by large‐bodied active and passive dispersers, such as fish and macrophytes (Padial et al., 2014), the four paradigms of metacommunity can be synthesized well in the context implemented by the classic spatially‐implicit SS‐ME model.

### 4.2 Variation partitioning and autocorrelation analysis to distinguish between niche‐based processes and ND

Previous studies have shown two major problems of variation partitioning based on canonical analysis: (1) lack of an original environmental variable to model the unimodal responses of species (Smith & Lundholm, 2010; Sokol et al., 2017), (2) Bias in estimation and statistical test of species–environment relationships based on environmental components when both species and environment are spatially structured (Peres‐Neto & Legendre, 2010). In this study, first‐ and second‐order orthogonal environmental variables were used to model the unimodal responses (Borcard et al., 2011). The statistical tests of species–environment relationships were based on pure environmental components.

When comparing the detailed changes of properties of the species with the variation of species abundance explained by environmental and purely spatial components in the simulation outcomes, the problems of variation partitioning were clearly shown. In the model communities with only one guild, functional uniqueness was zero; however, the relative proportion explained by the environmental component was positively biased in the linear environmental gradient, markedly at the maximum dispersal rate in areas packed within biogeographic units. The Type I error of the purely environmental component was inflated in these scenarios. In model communities with two or more guilds, the functional uniqueness of complementary simulations would be at its maximum at an intermediate scale. However, the relative proportion explained by the environmental component (Appendix S2a) and the power of the purely environmental component in the linear environmental gradient (Appendix S2b) were greatest at the maximum dispersal rate in areas packed within biogeographic units. In general, ND, PD, and weak species–environment associations were confused in the spatial structures found within and among patchy habitats. Contrasting species compositions may be observed between opposite sides of the model communities because of the approximate scales of three for *m* = 0.01, nine for *m* = 0.09, and 28 for *m* = 0.81. Orthogonal environmental variables were required to model nonlinear responses but increased the spurious correlations in a spatially structured environment, specifically under the anisotropy of the environmental structure.

However, this problem can be solved using a recently proposed method: constrained randomization of environmental variables conserving the original spatial structures and inter‐correlations in environmental variables (Clappe et al., 2018; Wagner & Dray, 2015). Constrained randomization of the PCNM variables with positive Moran's I values built from the environmental distance, not only the first‐ and second‐order orthogonal environmental variables, lead to an adjusted estimate and valid statistical test for assessing the influence of environmental filtering across scenarios (Appendices S3–S5). However, the spatial component of the hierarchical model was underestimated in the linear environmental gradient and was often insignificant at the maximum dispersal rate in areas packed within biogeographic units (Appendix S4b). The relative proportion explained by the pure spatial component of the hierarchical model markedly decreased in these scenarios (Appendices S4a and S5a). Constrained randomization of the species composition within the hierarchical guild structure resulted in an adjusted estimate and valid statistical test for assessing the spatial component of the hierarchical model. As well as approximating the relative proportion accounted for by the purely spatial component of the overall model across scenarios (Appendices S6 and S7). Appropriately applied environmental components can be used to assess the influence of environmental filtering. If the environmental rather than the purely environmental component is significant, identifying the hierarchical guild structure may allow assessments of the link between the theoretical models in each habitat type.

Variation partitioning based on canonical analysis was used to link field observations to one of four paradigms in the metacommunity (Cottenie, 2005) or ME with limited dispersal (Ng et al., 2009). A decision tree was constructed to link the presence of only significant purely spatial components to ND or PD, only significant purely environmental components to SS, and both significant purely spatial and purely environmental components to ME with high or limited dispersal. This approach assumes that the spatial structure is not a dispersal proxy and not strictly equivalent to ND, despite all environmental factors included in the analysis. However, the spatial structure may result from ND, PD, and weak species–environment associations, which is an assumption supported by this study. When identifying the hierarchical guild structure, however, the last pattern can be linked to a set of theoretical models in a metacommunity that operates simultaneously. In this case, identifying only the spatial structure resulting from weak species–environment association as ME with limited or high dispersal is better. Thus, the relative proportion explained by environmental components, environmental filtering, and SS can be interchangeable, and the link between theoretical models can be assessed for each habitat type. The sets of theoretical models include (1) SS and ND as characterized by the third hypothesis of this study, (2) SS and weak association with limited dispersal (i.e., ME with limited dispersal), (3) SS and PD, (4) SS and weak association with high dispersal (i.e., ME with high dispersal), and (5) SS, ND, PD, etc. Considering that ME with limited dispersal and ME with high dispersal do not operate simultaneously, there are a total of 11 sets of theoretical models (Appendix S8). For SS ND (1), the residents of each habitat type were species of a single guild. For the other sets (2–11), residents of some or all habitat types were species of two or more guilds, although the difference in functional species traits for the weak association may be undetectable.

The performance of autocorrelation analysis (Diniz‐Filho et al., 2012) was unexpected but interesting. ND always generates negative and significant patterns in areas comprising several independent biogeographic units, whereas environmental filtering generates negative and significant patterns in areas packed within a single biogeographic unit. Intuitively, phylogenetically closely related species were clumped, and ND may obscure significant patterns if the model communities are much more densely packed within a biogeographic unit. Furthermore, ND, through evolutionary dynamics, and PD may generate significant patterns in patchy habitats.

By identifying the hierarchical guild structure in field studies, autocorrelation analysis can be applied in a straightforward manner to disentangle the influence of evolutionary dynamics and PD. The analysis of functional ecology (e.g., Pillar & Duarte, 2010) is then applied to separate the influence of PD from ND and the weak species–environment associations, which may be confused in spatial structures. These methods may also indicate the existence of unknown yet significant environmental factors that were not accounted for in the analysis.

### 4.3 Explanation of complex patterns observed in the real world

A set of theoretical models operate simultaneously in a metacommunity to model real‐world conditions. In riverine systems, Brown and Swan (2010) argued that peripheral headwater communities would be structured primarily by SS, whereas central mainstream communities would be structured by ME examining macroinvertebrate communities. Schmera et al. (2018) referred to this as the network position hypothesis (NPH). By examining benthic diatoms, macrophytes, macroinvertebrates, and fish communities, they argued that large differences in the dispersal modes of organisms within and among focal taxa might affect the prediction of NPH. They found supporting evidence for NPH only for macroinvertebrate communities. Furthermore, the observed patterns differed when macroinvertebrate taxa were divided into flying and nonflying groups. The influence of environmental filtering may be greatest at the maximum dispersal rate for large‐bodied active dispersers. For intermediate dispersal rates for large‐bodied passive dispersers, these predictions seem to explain field observations in fish (i.e., SS and ND; SS and weak association with limited dispersal in the headwater; and SS in the mainstream) and macrophytes (i.e., SS in the headwater; and SS and weak association with high dispersal in the mainstream).

In the fish communities of the lowlands of the Bolivian Amazon, a hierarchical guild structure was observed across a range of spatiotemporal scales (Yunoki et al., 2017, 2018, 2019; Yunoki & Torres, 2016). These studies presumed that the spatial structures in patchy habitats only emerged from ND if all environmental factors were included in the analysis. In addition, ND did not generate significant patterns in the autocorrelation analysis and ND should not be associated with functional species traits. The final argument is based on neutral assumptions.

The temporal structures found in successional patchy habitats on a fine spatiotemporal scale usually presented autocorrelation patterns and were associated with seasonal and interannual changes in functional species traits. These patterns, along with important environmental factors that were missing from the analysis, were ascribed to environmental filtering.

The spatial structures found among patchy habitats on a very broad spatial scale presented an autocorrelation pattern in turbid rivers but not in varzea lakes. The spatial structures were associated with the divergence of species' ecological ranges in turbid rivers and transparent black‐clear water bodies but not in varzea lakes. The convergence of ecological ranges associated with hierarchical guild structures suggested that sedentary species were dominant in turbid rivers and transparent black‐clear water bodies. Autocorrelation patterns were found for sedentary species but not for migratory species. As migratory species were dominant in varzea lakes, these species did not present autocorrelation patterns. These patterns are interpreted as the natural selection of sedentary species in the stable environments of turbid rivers and transparent black‐clear water bodies, as the species might have adapted to a minor environmental factor missing from the analysis, and the ND of migratory species in the seasonal environment of varzea lakes (Yunoki et al., 2019).

However, the natural selection of sedentary species in turbid rivers and transparent black‐clear water is doubtful. Similar to nucleotide and amino acid substitution patterns in population genetics (Holsinger, 2015), natural selection may operate during the initial stage of diversification when the population size of each species is large and favorable attributes are conserved for their descendants. This process was approximated according to the mode of speciation and the range of the initial species pool in the system. The massive extinction of archaic fauna was triggered by climate change during the Paleogene. The roles of niche conservatism and environmental heterogeneity controlled by principal geomorphological features (e.g., water types) in the evolution of modern biodiversity during the Neogene were discussed by Albert and Reis (2011) in a synthesis of the biogeography of Neotropical freshwater fishes.

The general patterns observed in the simulations alter the interpretation of the field observations in this study and plausibly support the recent synthesis of biogeography. The spatial structures and autocorrelation patterns found among patchy habitats of turbid rivers and transparent black‐clear water bodies on a very broad spatial scale between the north and south of the Bolivian Amazon lowlands are associated with the divergence of fish communities. The autocorrelation patterns found for sedentary but not migratory species were explained by PD and the speciation of sedentary species with niche conservatism.

## 5 CONCLUSION

The niche‐based process is deterministic and results in a stationary state for ecologically‐different species. Simultaneously, ND is stochastic and eventually results in a dynamic equilibrium of ecologically‐equivalent species through a speciation–extinction balance. The dispersal modes of biota may affect how the influence of environmental filtering changes across ecological–evolutionary scales. For large‐bodied active dispersers, such as fish, the influence of environmental filtering may be greatest in compactly‐packed areas within biogeographic units. The species are filtered along the environmental gradient, and the coexistence of ecologically‐different species in each local community in a homogeneous environment is allowed by dispersals in a set of local communities. Both ND among the species of a single guild and PD among species with similar environmental optima and different levels of specialization and weak species–environment association (e.g., ME) operate simultaneously in patchy habitats. In spatially‐explicit synthesis, characterizing where a metacommunity falls along a niche‐neutral continuum is too simplistic and involves an abstraction that any biological process is probabilistic, therefore, dynamic–stochastic processes. The general patterns observed in the simulations allowed a theoretical synthesis of a metacommunity and helped explain the complex patterns observed in the real world.

## AUTHOR CONTRIBUTIONS

**Takayuki Yunoki:** Conceptualization (lead); formal analysis (lead); methodology (lead); software (lead); writing – original draft (lead); writing – review and editing (lead).

## CONFLICT OF INTEREST STATEMENT

I have no conflicts of interest to disclose.

## Supporting information


Appendix S1

Appendix S2

## Data Availability

The R sources are available at https://github.com/takayukiyunoki/spatialIBM. Appendix S1 presents the summary statistics of the scenarios presented in the main text. In Appendix S2, the summary statistics of the model communities that resulted in two or more guilds are compared across ecological and evolutionary scales in the three environmental structures. In simulations, the environmental niche only affected the per capita birth and death rates. In Appendices S3–S5, constrained randomization of environmental variables was applied for the scenarios presented in Figures 4 and 5 and Appendix S2. In Appendices S6 and S7, the constrained randomization of environmental variables and species composition within the hierarchical guild structure was applied for the scenarios presented in Figure 5 and Appendix S2. Appendix S8 presents 11 sets of theoretical models in a metacommunity. Our field data were publicly available from the Bolivian Amazon lowland fish metacommunity data. Freshwater Metadata Journal 7: 1–6. https://doi.org/10.15504/fmj.2015.7.
