# Supplementary material for: In a niche‐neutral continuum, a set of theoretical models in a metacommunity operates simultaneously in patchy habitats
Source: Ecol Evol. 2023 Feb 22;13(2):e9754. doi: 10.1002/ece3.9754 (PMC9943931; doi:10.1002/ece3.9754)
Supplement: Supplementary file 2 — Appendix S2 [file ECE3-13-e9754-s002.docx]

Appendix B Summary statistics of the model communities that resulted in at least two guilds were compared across ecological–evolutionary scales in three environmental structures. In simulations, the environmental niche only affected the per capita birth–death rates. The model parameters were the same as the scenarios presented in the main text, except for only one case of guild population size was explored. (a) Functional uniqueness and redundancy were compared to the relative proportions explained by environmental and pure spatial components in the total explained variation of the overall model; then, the total explained variation and these relative proportions were compared between overall and hierarchical models. The portions explained in the hierarchical model were calculated for the scenarios in that the hierarchical guild structure was significant. vs. guild-habitat, the number of guilds that coexisted in model communities was compared to the number of habitat types identified by hierarchical guild structure. (b) Power rate of all components in overall and hierarchical models. The number of model communities is provided within parentheses on the x-axis.


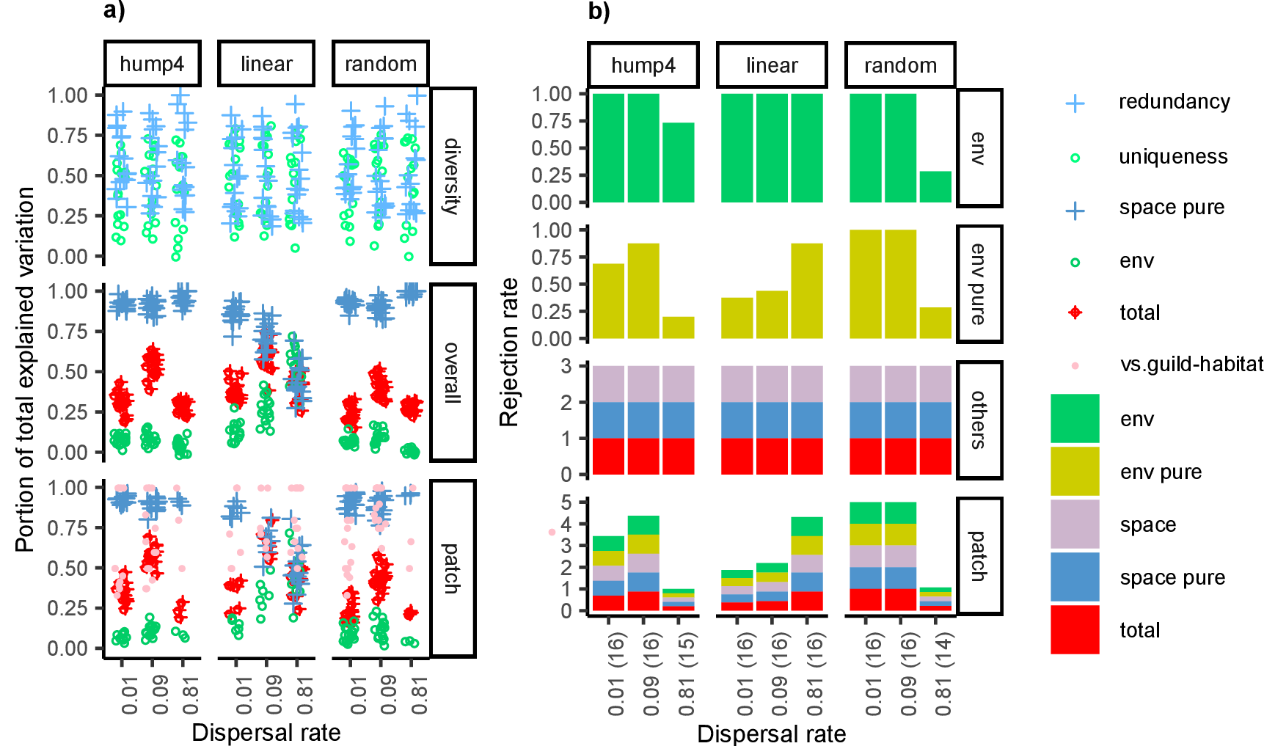


Appendix C Summary statistics of the model communities that resulted in only one guild were compared across ecological–evolutionary scales in three environmental structures. The constrained randomization of environmental variables: PCNM variables with positive Moran´s I values built from the environmental distance was applied for the scenarios presented in Figure 4. All environmental variables were used without forward selection. The replicates of environmental variables were generated using a spatial weight matrix defined by eight nearest neighboring communities. (a) Functional uniqueness and redundancy were compared to the relative proportions explained by environmental and pure spatial components in total explained variation. (b) Type I error rate of environmental and pure environmental components and power rate of other components. The number of model communities is provided within parentheses on the x-axis.


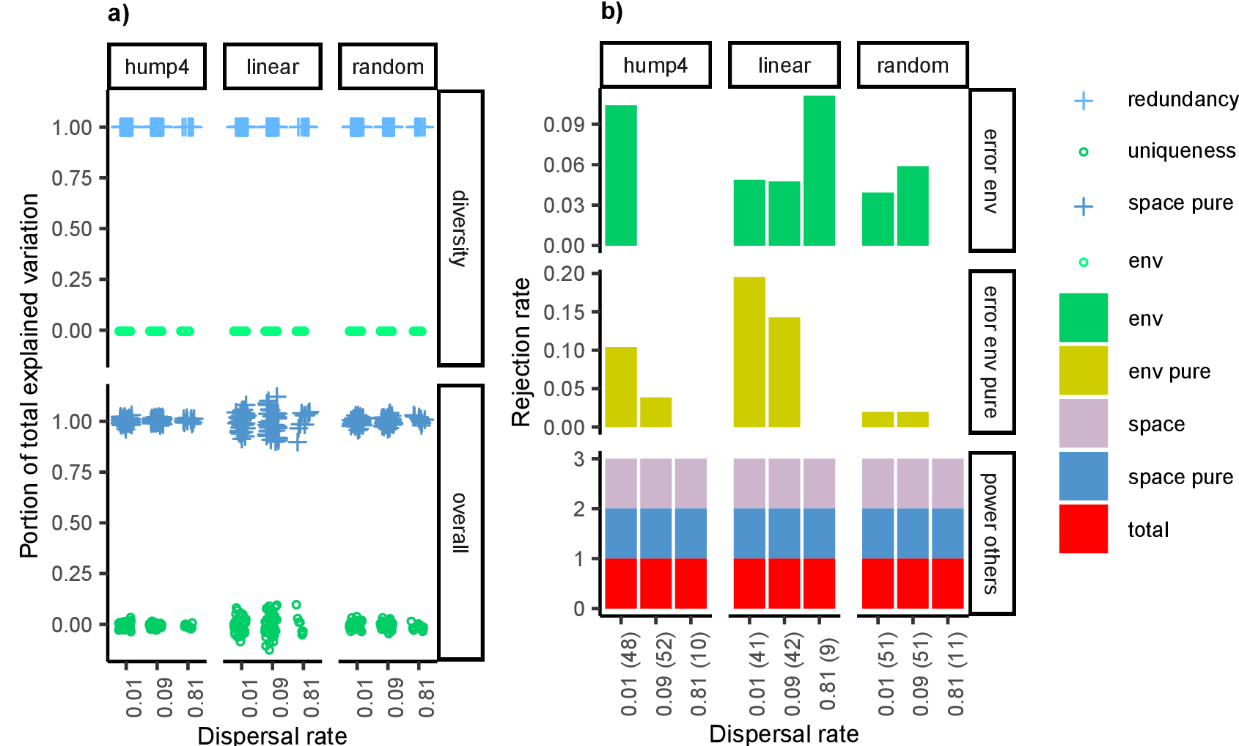


Appendix D Summary statistics of the model communities that resulted in at least two guilds were compared across ecological–evolutionary scales in three environmental structures. The constrained randomization of environmental variables: PCNM variables with positive Moran´s I values built from the environmental distance was applied for the scenarios presented in Figure 5. All environmental variables were used without forward selection. The replicates of environmental variables were generated using a spatial weight matrix defined by eight nearest neighboring communities. (a) Functional uniqueness and redundancy were compared to the relative proportions explained by environmental and pure spatial components in the total explained variation of the overall model; then, the total explained variation and these relative proportions were compared between overall and hierarchical models. The portions explained in the hierarchical model were calculated for the scenarios in that the hierarchical guild structure was significant. vs. guild-habitat, the number of guilds that coexisted in model communities was compared to the number of habitat types identified by hierarchical guild structure. (b) Power rate of all components in overall and hierarchical models. The number of model communities is provided within parentheses on the x-axis.


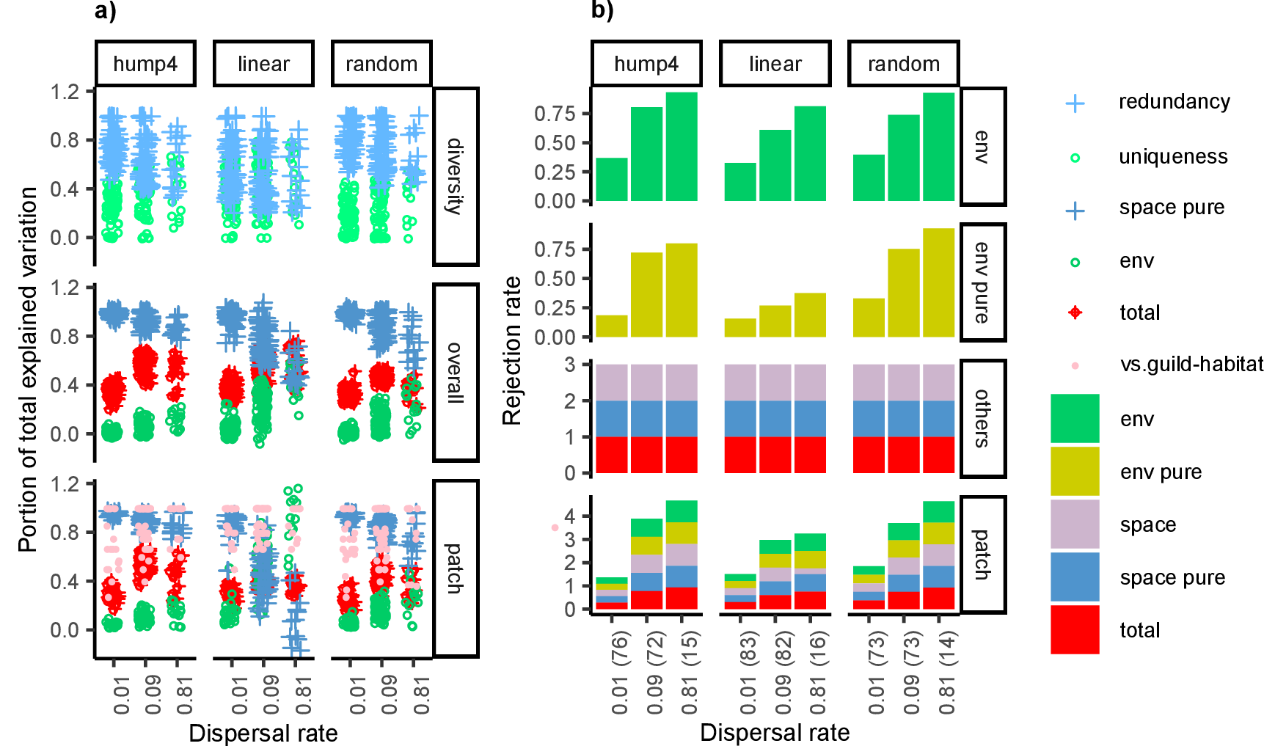


Appendix E Summary statistics of the model communities that resulted in at least two guilds were compared across ecological–evolutionary scales in three environmental structures. The constrained randomization of environmental variables: PCNM variables with positive Moran´s I values built from the environmental distance was applied for the scenarios presented in Appendix B. All environmental variables were used without forward selection. The replicates of environmental variables were generated using a spatial weight matrix defined by eight nearest neighboring communities. (a) Functional uniqueness and redundancy were compared to the relative proportions explained by environmental and pure spatial components in the total explained variation of the overall model; then, the total explained variation and these relative proportions were compared between overall and hierarchical models. The portions explained in the hierarchical model were calculated for the scenarios in that the hierarchical guild structure was significant. vs. guild-habitat, the number of guilds that coexisted in model communities was compared to the number of habitat types identified by hierarchical guild structure. (b) Power rate of all components in overall and hierarchical models. The number of model communities is provided within parentheses on the x-axis.


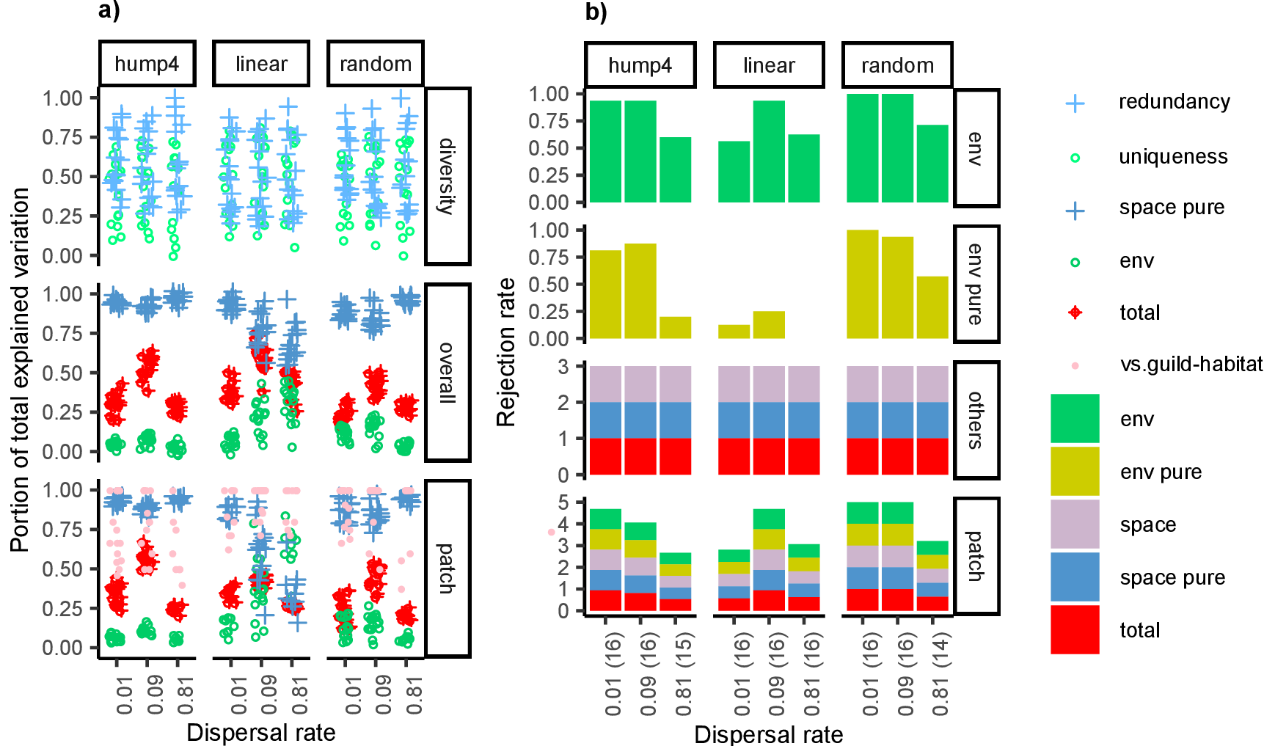


Appendix F Summary statistics of the model communities that resulted in at least two guilds were compared across ecological–evolutionary scales in three environmental structures. The constrained randomization of environmental variables: PCNM variables with positive Moran´s I values built from the environmental distance was applied for the scenarios presented in Figure 5. In this analysis, the simulation outcomes from only one case of guild population size were utilized. All environmental variables were used without forward selection. The replicates of environmental variables were generated using a spatial weight matrix defined by eight nearest neighboring communities. In addition, the randomization of species composition was constrained within a hierarchical guild structure. (a) Functional uniqueness and redundancy were compared to the relative proportions explained by environmental and pure spatial components in the total explained variation of the overall model; then, the total explained variation and these relative proportions were compared between overall and hierarchical models. The portions explained in the hierarchical model were calculated for the scenarios in that the hierarchical guild structure was significant. vs. guild-habitat, the number of guilds that coexisted in model communities was compared to the number of habitat types identified by hierarchical guild structure. (b) Power rate of all components in overall and hierarchical models. The number of model communities is provided within parentheses on the x-axis.


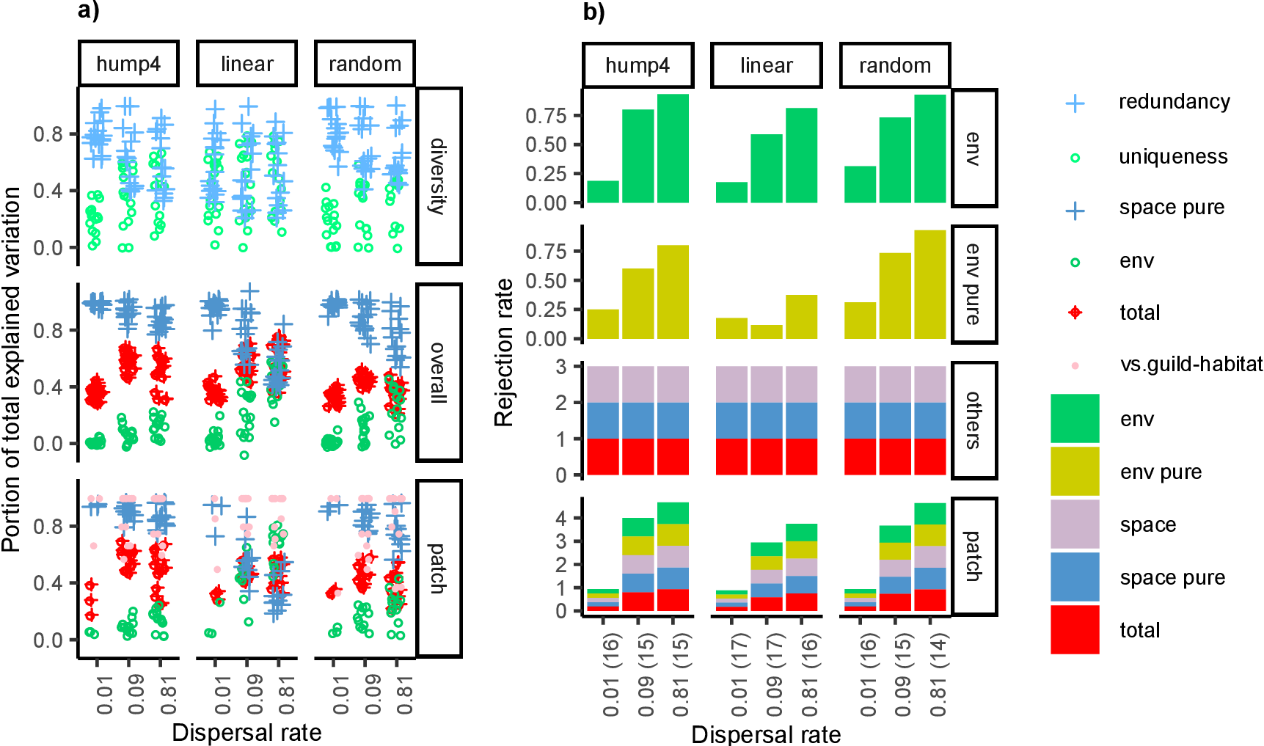


Appendix G Summary statistics of the model communities that resulted in at least two guilds were compared across ecological–evolutionary scales in three environmental structures. The constrained randomization of environmental variables: PCNM variables with positive Moran´s I values built from the environmental distance was applied for the scenarios presented in Appendix B. All environmental variables were used without forward selection. The replicates of environmental variables were generated using a spatial weight matrix defined by eight nearest neighboring communities. In addition, the randomization of species composition was constrained within a hierarchical guild structure. (a) Functional uniqueness and redundancy were compared to the relative proportions explained by environmental and pure spatial components in the total explained variation of the overall model; then, the total explained variation and these relative proportions were compared between overall and hierarchical models. The portions explained in the hierarchical model were calculated for the scenarios in that the hierarchical guild structure was significant. vs. guild-habitat, the number of guilds that coexisted in model communities was compared to the number of habitat types identified by hierarchical guild structure. (b) Power rate of all components in overall and hierarchical models. The number of model communities is provided within parentheses on the x-axis.


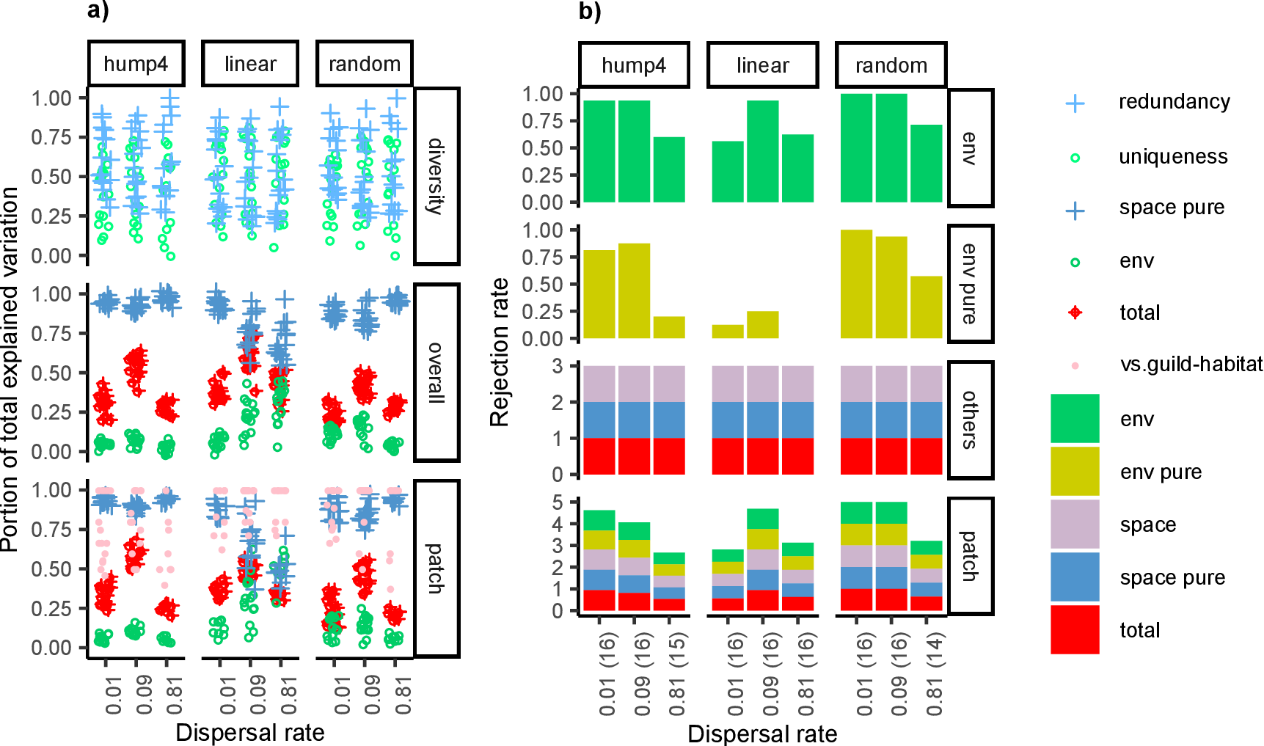


Appendix H Sets of theoretical models of metacommunity operating simultaneously in patchy habitats. SS: species sorting, ND: neutral dynamics, ML: mass effect with limited dispersal, PD: patchy-dynamics, MH: mass effect with high dispersal.
